# Systematic review and meta-analysis: effects of maternal separation on anxiety-like behavior in rodents

**DOI:** 10.1038/s41398-020-0856-0

**Published:** 2020-06-01

**Authors:** Daniel Wang, Jessica L. S. Levine, Victor Avila-Quintero, Michael Bloch, Arie Kaffman

**Affiliations:** 1 Department of Psychiatry, Yale University School of Medicine, 300 George Street, Suite 901, New Haven, CT 06511 USA; 2 Child Study Center, Yale University School of Medicine, 230 South Frontage Road, New Haven, CT 06519 USA

**Keywords:** Scientific community, Psychiatric disorders

## Abstract

The mechanisms by which childhood maltreatment increases anxiety is unclear, but a propensity for increased defensive behavior in rodent models of early life stress (ELS) suggests that work in rodents may clarify important mechanistic details about this association. A key challenge in studying the effects of ELS on defensive behavior in rodents is the plethora of inconsistent results. This is particularly prominent with the maternal separation (MS) literature, one of the most commonly used ELS models in rodents. To address this issue we conducted a systematic review and meta-analysis, examining the effects of MS on exploratory-defensive behavior in mice and rats using the open field test (OFT) and the elevated plus maze (EPM). This search yielded a total of 49 studies, 24 assessing the effect of MS on behavior in the EPM, 11 tested behavior in the OFT, and 14 studies provided data on both tasks. MS was associated with increased defensive behavior in rats (EPM: Hedge’s *g* = −0.48, *p* = 0.02; OFT: Hedge’s *g* = −0.33, *p* = 0.05), effect sizes that are consistent with the anxiogenic effect of early adversity reported in humans. In contrast, MS did not alter exploratory behavior in mice (EPM: Hedge’s *g* = −0.04, *p* = 0.75; OFT: Hedge’s *g* = −0.03, *p* = 0.8). There was a considerable amount of heterogeneity between studies likely related to the lack of standardization of the MS protocol. Together, these findings suggest important differences in the ability of MS to alter circuits that regulate defensive behaviors in mice and rats.

## Introduction

Childhood maltreatment is a heterogenous group of adversities that includes parental neglect/abuse, poverty, neighborhood violence, and bullying by peers^1,2^. In most cases, different types of adversities co-occur to increase the risk for developing multiple psychiatric and medical conditions in a dose-dependent manner^3–6^. Interventions that improve parental care in high-risk population lead to robust and sustained improvement in several behavioral and cognitive outcomes^7–11^, supporting a causal relationship between maltreatment and the presence of behavioral abnormalities later in life. One of the most robust clinical findings in individuals exposed to childhood maltreatment is a 2–3 fold increase in the odds-ratio for developing anxiety disorder^3,4,12–14^, including conditions such as specific phobias, social anxiety, and panic disorders that have prominent defensive features such as increased autonomic responses and avoidance^15^. The distinction between measurable defensive responses and subjective feelings of anxiety is important because these two experiences might be mediated by two distinct circuits^15^. Moreover, the defensive circuitry, unlike higher cortical processing of subjective experiences, is relatively conserved between humans and rodents^15^.

Exactly how adverse events early in life alter circuits that regulate anxiety and defensive behaviors is a difficult question to address in humans because of the inherent complexity of the adversities, genetic variability, and numerous additional variables that are difficult to control in clinical settings. Moreover, mechanistic studies that causally link structural and functional changes with alteration in anxiety or defensive responses are difficult, if not impossible, to conduct in humans^2,16^. In this regard, the conserved nature of defensive circuits between rodents and humans^15^ suggests that animal models can provide important details about the mechanisms by which early adversity alters circuits that regulate defensive behaviors in humans. This assertion is supported be a large body of work showing that early life stress (ELS) increases amygdala activation in response to threat in both humans and rodents^2^. Moreover, such translational work may clarify how alterations in connectivity between subcortical defensive circuits affect higher cortical areas (e.g., the prefrontal cortex, insula, and the parietal neocortex) that appear to regulate subjective perceptions of fear and anxiety^15^. For example, work by Ohashi et al. found that increased measures of amygdala connectivity is associated with increased symptomatology in humans, including anxiety^17^, and mice exposed to complex and unpredictable stress early in life show reduced time in the center of the open field test (OFT) and reduced time exploring the open arm in the elevated plus maze (EPM), both of which are measures of exploratory-defensive behaviors^15^. Importantly, this increase in defensive behavior was highly correlated with increased fronto-limbic connectivity including connections between the amygdala and the prefrontal cortex^18^. This is important because optogenetic activation of amygdala-prefrontal cortex connections increases defensive behavior in the OFT and EPM^19,20^. SSRIs and benzodiazepine which are the most effective pharmacological treatment for anxiety in humans, also decrease defensive behavior in rodents including exploratory behavior in the OFT and EPM^21^. Finally, work in rodents allows researchers to rigorously control the genetic background, standardize the stressors, and causally link developmental changes with alterations in defensive behavior^2^.

One of the most commonly used paradigm for ELS in rodents is maternal separation (MS). In this procedure, pups are separated for 1–6 h daily during the first 2–3 weeks of life^22–26^. MS was popularized in the early 90’s by studies showing that extended and repeated periods of maternal separation early in life caused long-term increase in stress reactivity^27–29^. This finding was intriguing because previous work has shown that short bouts of repeated maternal separation (e.g., 5–10 min), a manipulation known as handling or brief maternal separation, blunted stress reactivity and reduced defensive behavior^23^. Additional work has shown that prolonged periods of maternal absence is necessary to increases corticosterone and to alter growth hormone and overall activity in the pups^30–32^. This is consistent with the notion that altricial rodent pups are absolutely dependent on their mother for thermoregulation, nutrition, and protection^30–33^ and that MS represents a preclinical model of repeated threat early in life^2^.

Despite 3 decades of research with well over 100 publications including two recent systematic reviews (but not meta-analyses)^22,34^, the association between MS and defensive behavior remains unclear with some studies reporting significant increase in defensive behavior while others note no change or even reduced defensive behavior in rodents exposed to MS^2,22,34^. It is also unclear whether sex alters the effects of MS on defensive behavior and whether mice and rats are similarly affected. Additional unresolved methodological issues include whether isolating the pups from their littermates or exposing them to ambient temperature affect defensive behavior later in life. Finally, it is currently unclear whether some behavioral tests are more sensitive than others for detecting defensive response to MS.

To address these issues we conducted a systematic review and meta-analysis to examine the effects of MS on defensive-exploratory behavior in the OFT and EPM in mice and rats, two of the most commonly used anxiety-like behavior tests in rodents^35^. Meta-analysis is an approach commonly used in clinical research to synthesize evidence across multiple studies but has been rarely used in the animal literature^16^. In fact, we are aware of only one meta-analysis examining the effects of MS on pain sensitivity in rodents^36^, and no other examples of meta-analyses assessing psychiatrically relevant outcomes such as defensive behavior, reward devaluation, or vulnerability to substance abuse. The specific aims of this meta-analysis were to quantify the effect sizes of MS on exploratory-defensive behavior in the OFT and EPM and determine significance when compared to non-separated controls. The moderating effects of sex, species (rats vs, mice), length of separation from the dam, number of days pups were separated, temperature of isolation (ambient temp vs. incubator), single versus group isolation, and age of testing on outcomes in offspring exposed to MS were also examined. Finally, we assessed for potential publication bias and quantified the degree of heterogeneity in the literature.

## Methods

### Search strategy

Two reviewers (D.W. and J.L.) searched the electronic database of PubMed and Web of Science on April 1st, 2019 for relevant studies using the following search: (maternal separation) AND (mice OR mouse OR mus musculus OR rats OR rat) AND (elevated plus maze OR open field test). Studies were limited to English language studies. References of included studies and relevant reviewers were searched for additional citations. The titles and abstracts of the studies obtained through the search were examined by two reviewers (D.W. and J.L.) in order to determine article inclusion. Discrepancies were addressed by the reviewers through discussion and eventually conversation with the senior reviewers (A.K. and M.H.B.).

### Study selection

Upon preliminary inclusion, studies were read in their entirety to confirm that the inclusion criteria were met. If not, studies were excluded to obtain a final list of papers for inclusion into our meta-analysis. Eligibility for the meta-analysis were based on the following eight criteria: (1) pups were physically removed from their home cage and separated from their dams for 1–6 h daily for up to 21 days (PND21), beginning within the first three days of life (PND 0–3). (2) Dams had to be removed from the home cage and did not undergo any further stress during the separation procedure. (3) Studies were conducted in rodents (rats or mice) and (4) utilized the open field and/or elevated plus maze tests. (5) Studies included data separated by sex (6) and included a control group of either animal facility reared (AFR) or non-handled (NH) conditions. Studies utilizing early handling (EH) as the only control group were excluded. (7) Studies had to be peer-reviewed and published in English. (8) Sufficient information was available to calculate effect sizes. When insufficient data was presented in the original manuscript to calculate effect size or results were not stratified by sex authors were contacted to obtain additional data.

### Data extraction

Data collected on each article included year of publication, authors, rodent species and strain, sample size, types of outcomes tested (EPM, OFT, or both), temperature of separation (recorded as the mean value if presented as a range), presence of an incubator during separation, whether pups were separated individually or as a litter, length of separation in hours, the day on which EPM or OFT was tested, the duration of the MS procedure in days, and type of control used. Test outcomes were collected as mean and variance measure (SEM or SD) by sex and experimental group for each study. When these data were available only in graphical form, the program WebPlotDigitizer (Ankit Rohatgi, 2019) was utilized to convert graphically represented data into numerical values using the distance measurement function^37^. A detailed information with all variables is available in Table S1.

### Data analyses

All statistical analyses were completed in Comprehensive Meta-Analysis Version 3.0 (Biostat, 2016). Our primary outcomes of interest were indications of anxiety in the EPM and OFT tests. For example, time or percentage of time spent in the open arms of the EPM, or time spent of percentage of time spent in the center of the OFT, are commonly utilized indications of anxiety in MS studies. A random effects model was used as the primary method for meta-analysis for all primary outcomes and moderator analyses. Hedge’s *g* was used as the pooled measurement of effect size as it is preferred over Cohen’s *d* for small samples (which are common in animal studies)^38^. All primary results on the two outcomes of interest, OFT and EPM, are presented for the overall sample (stratified by sex and species) as well as for each individual sex (stratified by species). Publication bias was assessed using funnel plots and the Egger’s test. Heterogeneity was measured utilizing the *I*^2^ statistic and Chi-square test for heterogeneity^39,40^. The importance of these subgroups (sex and species) was examined utilizing the Chi-square test for subgroup differences^40^. A Random-effects model was utilized to assess the importance of moderators of interest using Comprehensive Meta-Analysis Version 3.0. Moderators of interest assessed included species, length of separation (hours), single versus whole litter separation, start of separation, duration of separation (days), age of testing, presence of an incubator during separation, temperature of separation procedure, and control type. All moderators were assessed individually after adjusting for species (and sex when necessary), the threshold of statistical significance was set at *p* < 0.05. Additionally, all moderators of interest were entered into a backward-stepwise regression model and eliminated based on overall significance level once co-linearity was accounted for in the analysis. When co-linearity was present in the model, backward-stepwise linear regression was conducted including each of the variables in separate backward-stepwise regression analysis. Potential moderators of interest were removed until all included variables had a threshold of statistical significance *p* < 0.1. Potential moderators with substantial collinearity (e.g., ambient temperature and use of incubator) did not present in any of the final models. Moderator analysis involved multiple hypothesis testing without appropriate statistical correction and should be regarded as exploratory for hypothesis-generating purposes in order to explain the large amount of heterogeneity present in these studies.

## Results

### Selection of studies

**Fig. 1:**
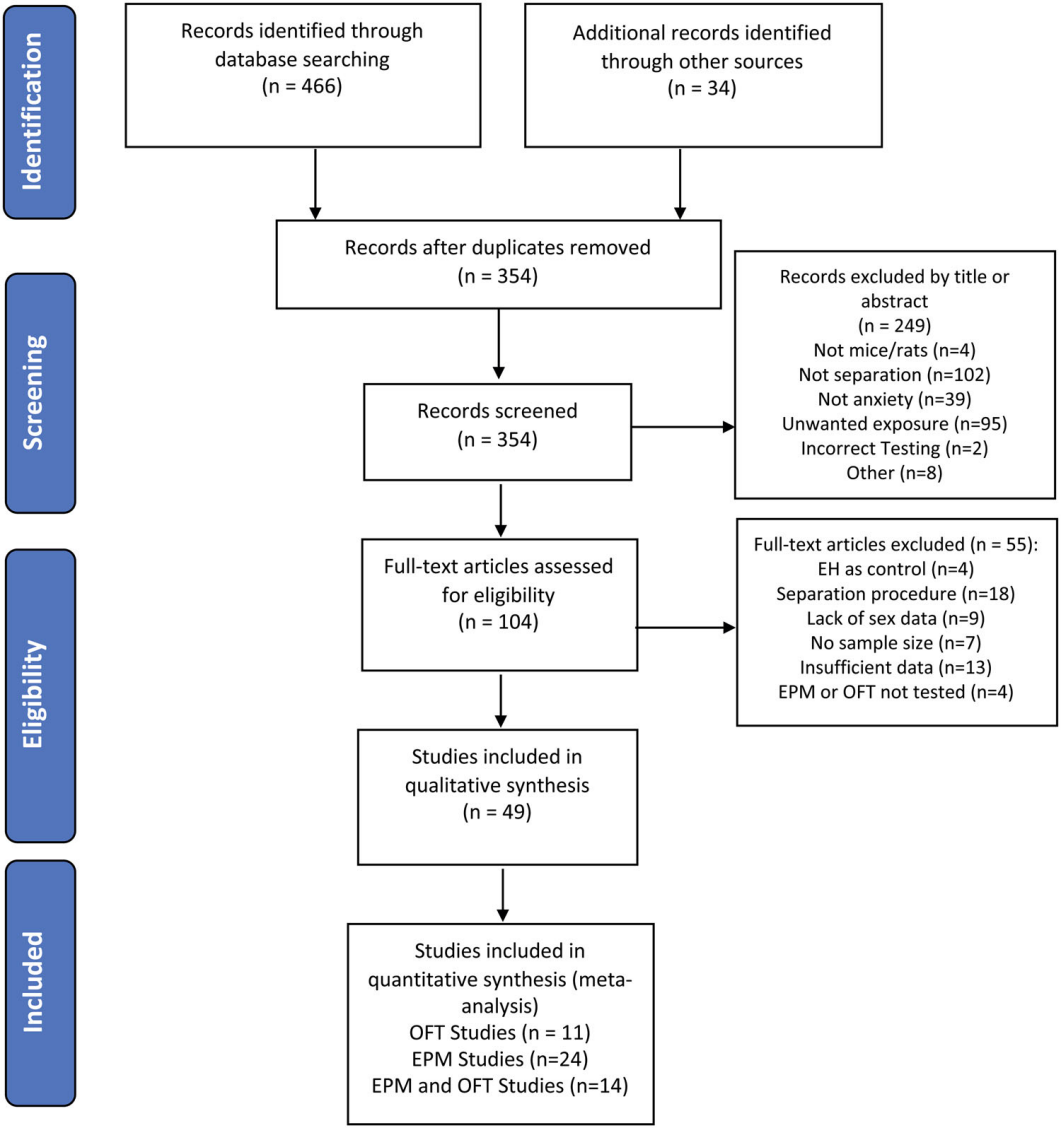
PRISMA flow diagram. A PRISMA flow diagram depicting selection of studies is shown.

Figure 1 depicts the selection strategy for included studies. Five hundred articles were identified for consideration in the present meta-analysis. Forty-nine studies were eligible for inclusion in this meta-analysis. Reasons for exclusion of studies are identified in Fig. 1 and “Methods” section. Table 1 describes the characteristics of our included studies and additional details are available in Table S1. Of the 49 included studies, 24 studies examined the effect of MS on behavior in the EPM, 11 studies tested behavior in the OFT, and 14 studies provided data on both tasks.

**Table 1:** List of studies included in the meta-analysis.

Reference	Species tested	Format of separation	Temp at separation	Age of testing	Sample size	Outcome(s) tested
Bondar^59^	Mouse- C57BL/6	P2–P12, 6 h/d	31 °C	P85	6–11/group	EPM, OFT
Clinton^60^	Rat- Sprague-Dawley	P1–P13, 3 h/d	37 °C	P75	12/group	EPM, OFT
Jaimes-Hoy^61^	Rat- Wistar	P2–P19, 3 h/d	31 °C	P40	8–10/group	EPM, OFT
Jin^62^	Rat- Wistar	P1–P20, 3 h/d	Not reported	P49	8–14/group	EPM, OFT
Markostamou^63^	Rat: Wistar	P1–P5, 3 h/d	31 °C	P90	2–11/group	EPM, OFT
Melo^54^	Rat: Wistar	P2–P12, 3 h/d	37 °C	P85	7–14/group	EPM, OFT
Millstein^41^	Mouse: 129S1; BALB/cByJ; C57BL/6j; DBA/2 J; FVB/NJ	P0–P13, 3 h/d	32 °C	P56	9–15/group	EPM, OFT
Nam^64^	Rat: WKY	P1–P13, 3 h/d	37 °C	P75	15/group	EPM, OFT
Roman^65^	Rat: Wistar	P1–P21, 6 h/d	27 °C	P73	15/group	EPM, OFT
Romeo^66^	Mouse: C57BL/6	P1–P13, 3 h/d	21 °C	P75 and P90	11–18/group	EPM, OFT
Savignac^67^	Mouse: C57BL/6; BALB/c	P1–P13, 3 h/d	Not reported	P63	15/group	EPM, OFT
Veenema^68^	Mouse: C57BL/6	P1–P13, 3 h/d	31.5 °C	P84	18–20/group	EPM, OFT
Weiss^69^	Mouse: C57BL/6j	P1–P13, 3 h/d	21 °C	P84	7–16/group	EPM, OFT
Xiong^70^	Rat: Sprague-Dawley	P2–P12, 3 h/d	21 °C	P56	8–9/group	EPM, OFT
Aisa^71^	Rat: Wistar	P2–P19, 3 h/d	Not reported	P60	10/group	EPM
Amini-Khoei^72^	Mouse: NMRI	P2–P12, 3 h/d	31.5 °C	P50	8/group	EPM
Bulbul^73^	Rat: Sprague-Dawley	P2–P12, 3 h/d	Not reported	P56	10–18/group	EPM
Dandi^74^	Rat: Wistar	P1–P20, 3 h/d	30 °C	P67	5–7/group	EPM
Eiland^75^	Rat: Sprague-Dawley	P2–10, 3 h/d	31 °C	P100	13/group	EPM
Grace^76^	Rat: Sprague-Dawley	P2–12, 3 h/d	24 °C	P49	11–13/group	EPM
Kalinichev^77^	Rat: Long Evans	P2–12, 3 h/d	31 °C	Not reported	8–12/group	EPM
Lajud^78^	Rat: Sprague-Dawley	P1–13, 3 h/d	31.5 °C	Not reported	13–14/group	EPM
Lee^79^	Rat: Sprague-Dawley	P1–13, 3 h/d	22 °C	P68	8/group	EPM
Lee^80^	Rat: Sprague-Dawley	P1–13, 3 h/d	21 °C	P56	8/group	EPM
Li^81^	Rat: Wistar	P1–20, 4 h/d	29 °C	P35	8/group	EPM
McIntosh^82^	Rat: Sprague-Dawley	P2–19, 3 h/d	32.5 °C	P35	10/group	EPM
Oines^83^	Rat: Wistar	P2–12, 3 h/d	31 °C	P66	18/group	EPM
Park^84^	Rat: Wistar	P3–11, 3 h/d	21 °C	P62	6/group	EPM
Plo^85^	Rat: Wistar	P1–20, 6 h/d	25 °C	P22	12/group	EPM
De Melo^86^	Rat: Long Evans	P3–12, 3 h/d	34 °C	P87	6–8/group	EPM
Rodriguez and Duenas^87^	Rat: Wistar	P1–20, 6 h/d	26.5 °C	P65	10–16/group	EPM
Ryu^88^	Rat: Sprague-Dawley	P1–13, 3 h/d	21 °C	P68	9/group	EPM
Slotten^89^	Rat: Long Evans	P3–12, 3 h/d	30 °C	P90	23/group	EPM
Sterley^90^	Rat: WKY	P2–12, 3 h/d	32 °C	P28	8–10/group	EPM
Venerosi^91^	Mouse: CD1	P2–12, 3 h/d	30 °C	P80	8–9/group	EPM
Wigger^92^	Rat: Wistar	P3–9, 3 h/d	37 °C	P112	8–9/group	EPM
Zhang^93^	Rat: Wistar	P2–13, 3 h/d	29 °C	P65	10/group	EPM
Zoicas^94^	Mouse: CD1	P1–13, 3 h/d	31.5 °C	P63	12/group	EPM
Aya-Ramos^95^	Rat: Wistar	P1–21, 6 h/d	Not reported	P51	7–10/group	OFT
Diehl^96^	Rat: Wistar	P1–9, 3 h/d	34 °C	P88	12/group	OFT
Ershov^97^	Mouse: C57BL/6	P2–14, 3 h/d	31 °C	P95	6–11/group	OFT
Kundakovic^98^	Mouse: BALB/c	P1–13, 2 h/d	Not reported	P35	10/group	OFT
Own^99^	Mouse: C57BL/6	P2–12, 3 h/d	31 °C	P70	29–30/group	OFT
Pierce^100^	Mouse: C57BL/6	P1–14, 3 h/d	34 °C	P42	5–6/group	OFT
Shalev^101^	Rat: Long Evans	P3–11, 3 h/d	32 °C	P77	15–16/group	OFT
Shu^102^	Rat: Sprague-Dawley	P2–12, 3 h/d	21 °C	P90	12/group	OFT
Stevenson^103^	Rat: Lister Hooded	P2–12, 6 h/d	31 °C	P90	11/group	OFT
Tsuda^104^	Mouse: C57BL/6	P1–13, 3 h/d	36 °C	P91	12–23/group	OFT
Tsuda^105^	Mouse: C57BL/6j	P1–13, 4 h/d	36 °C	P91	6–9/group	OFT

Postnatal age (P).

For additional details see Table S1 in the Supplemental information.

### Elevated plus maze (EPM)

**Fig. 2:**
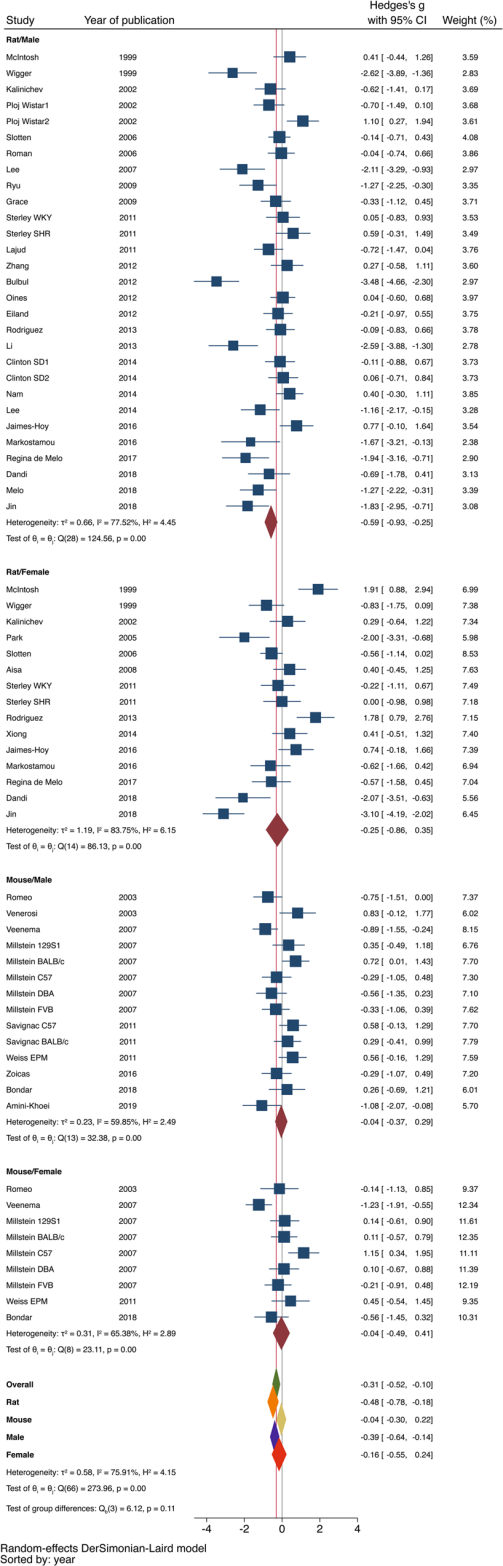
EPM forest plot. Forest plot assessing the effects of maternal separation on exploratory behavior in the EPM.

Figure 2 is a forest plot depicting the association between maternal separation and exploratory behavior in the 38 studies that examined this issue using the EPM (*n* = 1529 rodents). Some studies appear in Fig. 2 multiple times due to the fact that behavior was tested in males and females, and when using slightly different procedures of MS (e.g., Clinton SD1 vs. Clinton SD2), or different strains (e.g., Sterley WKY vs. Sterley SHR). The overall effect of maternal separation was significant in the EPM [Hedges *g* = −0.31 ± 0.11 (95% confidence interval (CI) = −0.52–(−0.10)), *z* = −2.85, *p* = 0.004, *k* = 67 treatment arms] with significant heterogeneity between studies (*I*^2^ = 76%, *Q* = 274, df = 66, *p* < 0.001). There was also significant evidence of publication bias as indicated by funnel plot asymmetry and the egger’s test (*p* = 0.001). When adjusting for funnel plot asymmetry using Duval and Tweedie’s Trim-and-Fill method, the association between maternal separation and elevated maze performance was no longer statistically significant Hedges *g* = −0.10 (95% CI) = −0.34–0.14).

**Fig. 3:**
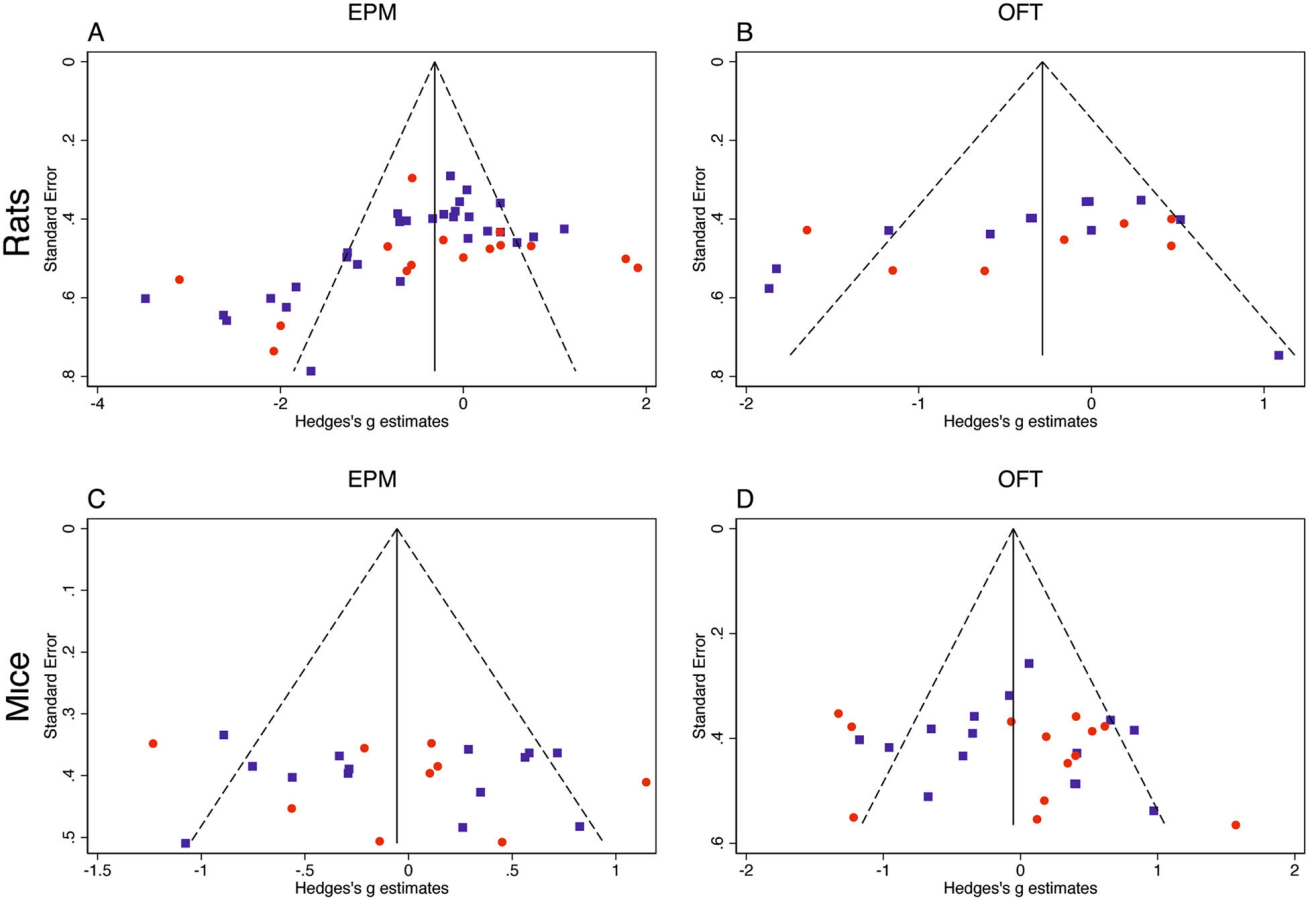
Funnel plots for EPM and OFT. Funnel plots of studies assessing the effects of maternal separation on exploratory behavior in the EPM (**a**, **c**) and OFT (**b**, **d**). Rats (**a**, **b**), mice (**c**, **d**). Males—blue squares, females—red circles.

Stratified subgroup analysis found a significant difference in the association between maternal separation and performance in the EPM based on species (*Q* = 4.67, df = 1, *p* = 0.03) but not sex (*Q* = 0.95, df = 1, *p* = 0.33). In rats, MS was associated with reduced time exploring the open arms of the EPM, a measure of defensive behavior (Hedges *g* = −0.48 ± 0.15, 95% CI = −0.78–(−0.18), *z* = −2.29, *p* = 0.02, *k* = 44). In contrast, mice exposed to MS showed similar exploratory behavior in the EPM compared to control group (Hedges *g* = −0.04 ± 0.13, 95% CI = −0.30–0.22, *z* = −0.32, *p* = 0.75, *k* = 23), see bottom of Fig. 2. There was significant evidence of publication bias among rat studies but not mice studies as indicated by the egger’s test (rat: *p* = 0.001 and mice *p* = 0.52) and by funnel plot asymmetry (Fig. 3a, c). When adjusting for funnel plot asymmetry using Duval and Tweedie’s Trim-and-Fill method, the association between maternal separation and performance in the EPM among rat studies was no longer statistically significant (Hedges *g* = −0.14 (95% CI) = −0.48–0.19). Males (Hedges *g* = −0.39 ± 0.13, 95% CI = −0.64–(−0.14), *z* = −3.00, *p* = 0.003, *k* = 43) but not females (Hedges *g* = −0.16 ± 0.20, 95% CI = −0.55–0.24), *z* = −0.78, *p* = 0.44, *k* = 24) showed significant increase in EPM anxiety, but with no significant effect of sex (Fig. 2).

Moderator analyses found significant effects of age of testing (*β* = 0.29 ± 0.14, 95% CI = 0.02–0.56, *z* = 2.13, p = 0.03) and length of separation (*β* = −0.011 ± 0.005, 95% CI = −0.02–(−0.0001), *z* = −1.96, *p* = 0.0497), after adjusting for the effects of species and sex. These findings indicated greater impact of maternal separation on exploratory behavior in the EPM in younger animals and those that were separated from the dam for longer periods. No significant effects were found for temperature of isolation, or single vs. whole litter isolation.

### Open field test (OFT)

**Fig. 4:**
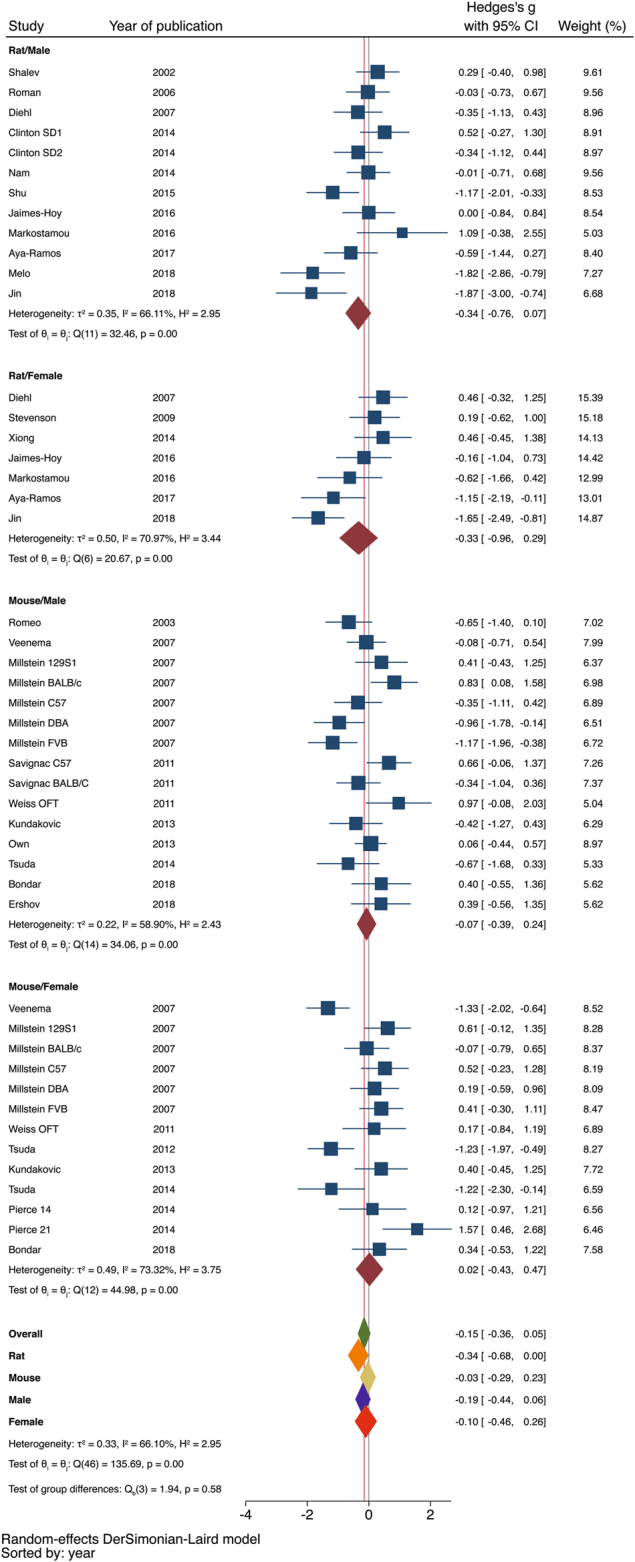
OFT forest plot. Forest plot examining the effects of maternal separation on exploratory behavior in the OFT.

Figure 4 is a forest plot depicting the effects of maternal separation on exploratory-defensive behavior in the OFT in the 24 studies included in the analysis (*n* = 1117 rodents). The overall effect of MS was not significant in the OFT (Hedges *g* = −0.15 ± 0.11, 95% CI = −0.36–0.05, *z* = −1.45, *p* = 0.15, *k* = 47 treatment arms). There was significant heterogeneity between studies (*I*^2^ = 66%, *Q* = 136, df = 46, *p* < 0.001) with no evidence of publication bias on the funnel plot or using the egger’s test (*p* = 0.62).

Stratified subgroup analysis did not find significant effects of species (*Q* = 1.98, df = 1, *p* = 0.16) or sex (*Q* = 0.16, df = 1, *p* = 0.70). However, similar to the findings in the EPM, maternal separation reduced exploration of the center of the OFT in rats (Hedges *g* = −0.34 ± 0.17, 95% CI = −0.67–(−0.00003), *z* = −1.96, *p* < 0.05, *k* = 19), but not mice (Hedges *g* = −0.03 ± 0.13, 95% CI = −0.29–0.12, *z* = −0.25, *p* = 0.80, *k* = 28), see bottom of Fig. 4. There was no evidence of publication bias in either rat or mice studies on the funnel plot (Fig. 3b, d) or using the egger’s test (rat: *p* = 0.17 and mice *p* = 0.51). No significant effects of MS on exploratory behavior in the OFT were found when males (Hedges *g* = −0.19 ± 0.13, 95% CI = −0.44–0.06, *z* = −1.46, *p* = 0.14, *k* = 27) and females (Hedges *g* = −0.10 ± 0.18, 95% CI = −0.46–0.26, *z* = −0.55, *p* = 0.59, *k* = 20) were assessed separately. No significant moderating effects were found for age of testing, length of separation, temperature of isolation, or single vs. whole litter isolation.

## Discussion

This is the first meta-analysis investigating the effects of early life stress on psychiatrically relevant behavioral outcomes in animals. Given the robust increase in anxiety and defensive behaviors seen in clinical setting^3,4,12–14^, we focused on the effects of MS, one of the most commonly used paradigms of early life stress in rodents, on defensive-exploratory behavior in the EPM (*n* = 1529) and OFT (*n* = 1117). One of the most intriguing finding of our analysis is that MS causes a significant increase in defensive-exploratory behavior in rats, but not in mice. This outcome was seen in both the EPM (Fig. 2) and the OFT (Fig. 4), with moderate effect sizes in both tests (rats EPM: Hedge’s *g* = −0.48, *p* = 0.02; rats OFT: Hedge’s *g* = −0.33, *p* = 0.05). In contrast, the effect sizes seen in mice were 10-fold smaller and were not statistically significant compared to control group (mice EPM: Hedge’s *g* = −0.04, *p* = 0.75; mice OFT: Hedge’s *g* = −0.03, *p* = 0.8). This finding substantiate previous anecdotical reports of difficulty establishing robust defensive-exploratory outcomes in mice exposed to MS^41–44^. However, this finding appears inconsistent with a recent systematic review by Tractenberg et al. that found overall increase in defensive-exploratory behavior in the majority of studies that examined this issue in mice^22^. This discrepancy is likely due to the use of weighted-combined effect sizes in this meta-analysis versus a more qualitative assessment based on the number of studies that reported increased defensive outcomes used by Tractenberg and colleagues. Moreover, the exact composition of studies is also somewhat different between our analysis and the systematic review conducted by Tractenberg. For example, we exclusively focused on studies using EPM and OFT while Tractenberg included other tests such as the dark-light test and social behavior. Different definitions of maternal separation were also used. For example, in this report MS had to be initiated during the first 3 days of life, and pups and dam must be removed from the home cage with no exposure to additional maternal stress, whereas in the Tractenberg review MS could be initiated after postnatal day 3, pups and/or dam could be left in home cage, and additional maternal stress was accepted. These differences highlight the lack of standardization that has plagued the MS paradigm for the past three decades.

The observations that rats and mice respond differently to MS is not surprising given that these two species diverged around 15–20 million year ago, an evolutionary gap that resembles the distance between macaque monkeys and the great apes, including *Homo Sapiens*^45^. Further, mounting evidence indicates that mice are not simply “smaller rats” and that there are important differences between these two commonly used rodent species. Some examples include significant changes in synaptic composition, serotonergic innervation, and levels of neurogenesis^45^. Perhaps most relevant to the question at hand is that rats appear to be more social than mice including their pet-like interaction with humans^45^. This increase in social complexity may affect circuits that regulate attachment rendering rat pups and dams more sensitive to separation. Moreover, the ability of rats to more easily habituate to human contact may also help reduce erratic behavior in tests of anxiety such as the EPM or the OFT.

Given the important role that maternal behaviors such as licking and grooming (LG) or arched-back nursing (ABN) play in the development of circuits that regulate defensive-exploratory behavior^23^ it is important to consider the effects of MS on these behaviors in rats and mice. This issue was recently reviewed by Orso et al. who found that MS induces higher levels of LG-ABN in rats compared to mice^46^. This increase in maternal behavior would expect to minimize and not to exacerbate the consequences of MS in rats. One possible explanation for this apparent discrepancy is to assume that the increase in maternal care seen in rats reflects higher levels of distress and vocalization induced by MS in rat pups compared to mice pups. Greater distress in rat pups might be associated with more significant developmental changes that are not reversed the compensatory increase in maternal care.

Our findings do not mean that mice are not suitable species for studying the effects of ELS on neurodevelopment or behavior, but rather that their use in the traditional MS paradigm for studying defensive-exploratory behaviors is challenging and will likely require some modifications. For example, mice exposed to MS early in life show enhanced defensive behaviors when exposed to social defeat in adulthood^47^, suggesting that a second stressor in adulthood might be necessary to induce robust defensive behaviors in mice exposed to MS. Moreover, mice exposed to limited bedding/nesting and unpredictable schedule of maternal separation show robust increase in defensive-exploratory behaviors in the OFT and EPM^18^.

The moderate effect size of maternal separation on defensive-exploratory behavior in rats (Hedges *g* = −0.33 to −0.48) is consistent with effect sizes reported for rates of anxiety disorder in humans exposed to childhood maltreatment (i.e., odds ratio of 2–3^3,4,12–14^ is roughly equivalent to effect sizes of 0.38–−0.6^48^). In addition, assuming an effect size of −0.4 in rats, a sample size of roughly 100 rats per group, is needed to ensure a power of 0.8 and α = 0.05^49^. This sample size is roughly 5–10 fold larger than the sample sizes commonly used in rodent work, suggesting that under-powered studies may contribute to the significant variance associated with behavioral outcomes in both the EPM (*I*^2^ = 76%, *Q* = 274, df = 66, *p* < 0.001) and the OFT (*I*^2^ = 66%, *Q* = 136, df = 66, *p* < 0.001). The large variability has been noted previously by others^22,26,50,51^ and is likely due to many factors including the lack of standardized procedure for conducting maternal separation, under-powered sample sizes. Further, the vulnerability of the EPM/OFT tests to numerous variables such as light/dark cycle, degree of illumination in the arena, sex of the tester, noise, smells, and order of behavioral testing^22,26,50,51^, highlights the need to develop more robust behavioral tests that rely on ambiguous-threatening cues, see refs. ^52,53^ for some encouraging examples.

The importance of standardizing the MS procedure is consistent with our findings that the length of separation (*β* = 0.29, *p* = 0.03) and earlier age of testing (*β* = −0.01, *p* = 0.0497) seem to impact exploratory behavior in the EPM. Interestingly, we found no significant effects of sex or the temperature during the separation on measures of anxiety in either the EPM or OFT. This is somewhat surprising, given previous reports that males appear more sensitive to the effects of MS compared to female rodents^2,34^, and that rodent pups are highly sensitive to ambient temperature^54–56^. However, given the large variability and the relatively small number of studies that have used ambient temperature, these issues will need further replication and clarification.

Significant funnel plot asymmetry and the egger’s test (*p* = 0.001) were found in studies utilizing the EPM in rats (Fig. 3a). These findings raise the possibility that the significant effect of MS on anxiety-like behavior in the EPM might be driven by a publication bias (i.e., the tendency not to publish negative results). We do not believe this is the case because similar effect size is seen in the OFT (Fig. 4) where no publication bias is seen (Fig. 3b). Moreover, Funnel Plot asymmetry can occur in the absence of a publication bias^57^. This is especially true in analysis with large variability, using under-powered samples, and relatively few studies, such as in our case^57,58^. Moreover, the reported sample sizes reflect number of rats used and do not reflect the number of independent litters used in each sample. This is important because pups from the same litter are not fully independent^2,26^ and, therefore, number of litters rather than total sample size may better reflect precision and position in the funnel plot.

In conclusion, our analysis highlights three important findings. First, MS alters exploratory behavior in the OFT and EPM in rats, but not mice, suggesting that circuits that regulate these defensive behaviors are affected differently by MS in these two species. Second, the effect size of maternal separation on these two behavioral tests in rats is roughly similar to the anxiogenic effect of early adversity reported in humans. These findings should not be interpreted to mean that outcomes of MS in rats better reflect changes seen in humans exposed to early diversity, but rather a call for more mechanistic studies that compare the effects of MS on circuits that regulate defensive-exploratory behaviors in these two species. Third, there is extensive variability among studies that needs to be addressed by better standardizing the maternal separation procedure and dramatically increasing the sample size.

## Supplementary information

Table S1
