# Supplementary material for: Systematic review and meta-analysis: effects of maternal separation on anxiety-like behavior in rodents
Source: Transl Psychiatry. 2020 Jun 1;10:174. doi: 10.1038/s41398-020-0856-0 (PMC7264128; doi:10.1038/s41398-020-0856-0)
Supplement: Supplementary file 1 — Table S1 [file 41398_2020_856_MOESM1_ESM.pdf]

Reference	ID	Species-strain	length of separation (1-6hr/d)	Start of Separation Period (PND)	Duration of Separation Period (Days)	Incubator vs Ambient Temp	Temperature (Celsius)	Single vs Whole Litter Separation	Age of testing (PND)	Sex	Control Type	Test	Outcome	Variance Measure	Mean	VM	n	Mean	VM	n	Mean	VM	n	mean	vm	n
Sterley et al. 2011	Sterley 2011 WKY	Rat –WKY	3	2	12	Incubator	32	Whole litter	28	M, F	AFR	EPM	% time spent in open arms vs open+closed	SEM	22.21	5.03	9	23.61	11.22	9	27.7	7	10	23.44	4.44	8
	Sterley 2011 SHR	Rat- SHR	3	2	12	Incubator	32	Whole litter	28	M, F	AFR	EPM	% time spent in open arms vs open+closed	SEM	14.78	4.4	9	21.83	3.11	9	35.08	6.22	5	35.08	4.34	14
Park et al. 2005	Park 2005	Rat- Wistar	3	3	11	Ambient Temp	21	Whole litter	62	F	AFR	EPM	% time spent in open arms vs open+closed	SEM	N/A	N/A	N/A	N/A	N/A	N/A	34.77	3.67	6	18.9	2.11	6
Rodriguez and Duenas 2013	Rodriguez 2013	Rat- Wistar	6	1	20	Incubator	26.5	Whole litter	65	M, F	AFR	EPM	Time spent in open arms (s)	SEM	6.18	3.1	16	5.16	2.75	11	21.64	3.95	10	53.77	6.27	11
Xiong et al. 2014	Xiong 2014	Rat- Sprague-Dawley	3	2	12	Ambient Temp	21	Whole litter	56	F	AFR	EPM	% time spent in open arms	SEM	N/A	N/A	N/A	N/A	N/A	N/A	8.23	2.83	8	12.29	3.51	9
Romeo et al. 2003	Romeo 2003	Mouse- C57BL/6	3	1	13	Ambient Temp	21	Whole litter	90 Males, 75 Females	M, F	AFR	EPM	Open arm vs total arm entry ratio (%)	SEM	40.11	3.93	11	32.12	1.96	18	15.05	4.97	6	12.82	5.91	8
Veenema et al. 2007a	Veenema 2007	Mouse- C57BL/6	3	1	13	Incubator	31.5	Whole litter	84	M, F	AFR	EPM	% time spent in open arms	Not Stated	14.55	2.53	18	7.15	1.05	20	22.32	3.14	20	8.88	1.08	18
Slotten et al. 2006	Slotten 2006	Rat- Long Evans	3	3	12	Incubator	30	Whole litter	90	M, F	NH	EPM	% time spent in open arms	SEM	15.5	2.6	23	14	1.7	23	28.6	2.6	23	21.9	2.3	23
Kalinichev et al. 2002	Kalinichev 2002	Rat- Long Evans	3	2	12	Incubator	31	Whole litter	N/A	M, F	NH	EPM	number of entries into closed arms	SEM	7.91	0.68	12	9.59	0.82	12	10.19	1.64	8	11.76	1.96	8
Bondar 2018	Bondar 2018	Mouse- C57BL/6	3	2	12	Incubator	31	Whole litter	85	M, F	AFR	EPM	% time spent in open arms	SEM	9.49	2.11	11	11.6	3.61	6	5.12	2.11	11	1.81	0.9	8
Regina de Melo 2017	Regina de Melo 2017	Rat- Long Evans	3	3	12	Incubator	34	Whole litter	87	M, F	AFR	EPM	Time spent in open arms (s)	SEM	23.5	6.76	6	1.12	0.78	8	17.33	9.9	6	7.12	2.86	8
Lee 2007	Lee 2007	Rat- Sprague-Dawley	3	1	13	Ambient Temp	22	Whole litter	68	M	NH	EPM	Time spent in open arms (s)	SEM	107.61	14.03	8	34.57	8.48	8	N/A	N/A	N/A	N/A	N/A	N/A
Clinton 2014	Clinton 2014 SD1	Rat- Sprague-Dawley	3	1	13	Incubator	37	Whole litter	75	M	AFR	EPM	% time spent in open arms	SEM	12.04	1.4	12	11.42	1.78	12	N/A	N/A	N/A	N/A	N/A	N/A
	Clinton 2014 SD2	Rat- Sprague-Dawley	3	1	13	Incubator	37	Whole litter	75	M	AFR	EPM	% time spent in open arms	SEM	5.91	0.67	12	6.16	1.42	12	N/A	N/A	N/A	N/A	N/A	N/A
Zhang 2012	Zhang 2012	Rat- Wistar	3	2	13	Incubator	29	Single	65	M	AFR	EPM	time spent in open arms (s)	SEM	80.7	5.5	10	86.2	6.96	10	N/A	N/A	N/A	N/A	N/A	N/A
Jaimes-Hoy 2016	Jaimes-Hoy 2016	Rat- Wistar	3	2	19	Incubator	31	Whole litter	40	M, F	AFR	EPM	time spent in open arms (s)	SEM	32.5	5	10	51	9	10	43	10	8	68	11	10
Dandi 2018	Dandi 2018	Rat- Wistar	3	1	20	Incubator	30	Whole litter	67	M, F	AFR	EPM	% time spent in open arms	SD	21.169	16.16255	5	11.8344	9.31514	7	34.0439	4.64856	5	15.5989	10.3716	5
Roman 2006	Roman 2006	Rat- Wistar	6	1	21	Incubator	27	Whole litter	73	M	AFR	EPM	time spent in open arms (s)	SEM	35	7	15	34	5	15	N/A	N/A	N/A	N/A	N/A	N/A
Lajud 2011	Lajud 2011	Rat- Sprague-Dawley	3	1	13	Incubator	31.5	Whole litter	N/A	M	AFR	EPM	time spent in open arms (s)	SEM	26.6	2.7	13	18.8	3	14	N/A	N/A	N/A	N/A	N/A	N/A
Millstein 2007	Millstein 2007 129S1	Mouse- 129S1/SvImJ	3	0	13	Incubator	32	Single	56	M, F	AFR	EPM	% time spent in open arms	SEM	11.23	4.64	12	16.52	4.13	9	20.65	2.35	11	22.75	4.58	15
	Millstein 2007 BALB/c	Mouse- BALB/cByJ	3	0	13	Incubator	32	Single	56	M, F	AFR	EPM	% time spent in open arms	SEM	5.87	2.46	14	12.32	2.03	17	8.5	2.22	18	9.8	3.66	14
	Millstein 2007 C57	Mouse- C57BL/6J	3	0	13	Incubator	32	Single	56	M, F	AFR	EPM	% time spent in open arms	SEM	11.16	3.12	12	8.62	1.59	13	9.28	1.57	16	16.41	1.7	11
	Millstein 2007 DBA	Mouse- DBA/2J	3	0	13	Incubator	32	Single	56	M, F	AFR	EPM	% time spent in open arms	SEM	18.91	2.32	15	13.91	2.54	10	14.91	1.96	9	15.85	2.35	18
	Millstein 2007 FVB	Mouse- FVB/NJ	3	0	13	Incubator	32	Single	56	M, F	AFR	EPM	% time spent in open arms	SEM	38.33	3.33	11	35.29	1.67	20	39.28	2.22	18	37.45	1.83	13
Ploj 2002	Ploj 2002 Wistar1	Rat- Wistar	6	1	20	Ambient Temp	25	Whole litter	22	M	AFR	EPM	time spent in open arms (s)	SEM	93.6	15.12	12	57.3	13.9	12	N/A	N/A	N/A	N/A	N/A	N/A
	Ploj 2002 Wistar2	Rat- Wistar	6	1	20	Ambient Temp	25	Whole litter	61	M	AFR	EPM	time spent in open arms (s)	SEM	80.6	14.4	12	144.7	17.8	12	N/A	N/A	N/A	N/A	N/A	N/A
Melo 2018	Melo 2018	Rat- Wistar	3	2	12	Incubator	37	Whole litter	85	M	AFR	EPM	% time spent in open arms	SEM	38.15	1.69	7	24.99	3.12	14	N/A	N/A	N/A	N/A	N/A	N/A
Savignac 2011	Savignac 2011 C57	Mouse- C57BL/6	3	1	13	Incubator	N/A	Single	77	M	AFR	EPM	% time spent in open arms	SEM	0.94	0.44	15	1.96	0.44	15	N/A	N/A	N/A	N/A	N/A	N/A
	Savignac 2011 BALB/c	Mouse- BALB/c	3	1	13	Incubator	N/A	Single	77	M	AFR	EPM	% time spent in open arms	SEM	0.19	0.05	15	0.35	0.19	15	N/A	N/A	N/A	N/A	N/A	N/A
Bulbul 2012	Bulbul 2012	Rat- Sprague-Dawley	3	2	12	Incubator	N/A	Whole litter	56	M	AFR	EPM	% time spent in open arms	SEM	29.7	2.9	10	6.8	1	18	N/A	N/A	N/A	N/A	N/A	N/A
Jin 2018	Jin 2018	Rat- Wistar	3	1	20	N/A	N/A	Not stated	49	M, F	AFR	EPM	Time spent in open arms (s)	SDM	39.63	11.4	8	15.7	13.27	8	36.36	9.35	14	11.03	6.17	14
Amini-Khoei 2019	Amini-Khoei 2019	Mouse- NMRI	3	2	12	Incubator	31.5	Whole litter	50	M	AFR	EPM	% time spent in open arms	SEM	12.7	1.28	8	8.99	1.01	8	N/A	N/A	N/A	N/A	N/A	N/A
Nam 2014	Nam 2014	Rat –WKY	3	1	13	Incubator	37	Whole litter	75	M	AFR	EPM	Time spent in open arms (s)	SEM	1.4	0.53	15	3.24	1.53	15	N/A	N/A	N/A	N/A	N/A	N/A
Lee 2014	Lee 2014	Rat- Sprague-Dawley	3	1	13	Ambient Temp	21	Whole litter	56	M	AFR	EPM	Time spent in open arms (s)	SEM	51.51	8.56	8	25.83	6.04	8	N/A	N/A	N/A	N/A	N/A	N/A
Li 2013	Li 2013	Rat- Wistar	4	1	20	Incubator	29	Single	35	M	AFR	EPM	Time spent in open arms (s)	SEM	98.55	6.73	8	43.24	7.53	8	N/A	N/A	N/A	N/A	N/A	N/A
Ryu 2009	Ryu 2009	Rat- Sprague-Dawley	3	1	13	Ambient Temp	21	Whole litter	68	M	AFR	EPM	Time spent in open arms (s)	SEM	38.67	11.35	9	5.67	2.52	9	N/A	N/A	N/A	N/A	N/A	N/A
Grace 2009	Grace 2009	Rat- Sprague-Dawley	3	2	12	Ambient Temp	24	Whole litter	49	M	AFR	EPM	Time spent in open arms (s)	SEM	38.58	4.48	13	33.8	3.09	11	N/A	N/A	N/A	N/A	N/A	N/A
Oines 2012	Oines 2012	Rat- Wistar	3	2	12	Incubator	31	Whole litter	66	M	NH	EPM	% time spent in open arms	SEM	18.9	4	18	19.5	2.8	18	N/A	N/A	N/A	N/A	N/A	N/A
Aisa 2008	Aisa 2008	Rat- Wistar	3	2	19	Incubator	N/A	Whole litter	60	F	AFR	EPM	Time spent in open arms (s)	SEM	N/A	N/A	N/A	N/A	N/A	N/A	33.67	4.76	10	41.58	6.99	10
Eiland 2012	Eiland 2012	Rat- Sprague-Dawley	3	2	10	Incubator	31	Whole litter	100	M	AFR	EPM	Time spent in open arms (s)	SEM	25.98	4.64	12.5	22.38	4.58	12.5	N/A	N/A	N/A	N/A	N/A	N/A
Markostamou 2016	Markostamou 2016	Rat- Wistar	3	1	5	Incubator	31	Whole litter	90	M, F	AFR	EPM	% time spent in open arms	SD	24.3565	11.09153	11	5.0279	7.11057	2	23.1066	16.48459	7	12.9769	13.56486	6
McIntosh et al. 1999	McIntosh et al. (1999)	Rat- Sprague Dawley	3	2	19	Incubator	32.5	Single	35	M, F	NH	EPM	% time spent in open arms	SEM	6.9	1.58	10	9.84	2.66	10	4.17	1.08	10	18.46	3.02	10
Zoicas 2016	Zoicas 2016	Mouse- CD1	3	1	13	Incubator	31.5	Whole litter	63	M	AFR	EPM	% time spent in open arms	SEM	25.69	2.85	12	23.21	1.76	12	N/A	N/A	N/A	N/A	N/A	N/A
Wigger 1999	Wigger 1999	Rat- Wistar	3	3	9	Incubator	37	Whole litter	112	M. F	AFR	EPM	% time spent in open arms	SEM	27.52	4.2	8	3.75	1.3	9	31.55	4.58	9	19.12	4.96	9
Weiss 2011	Weiss 2011 EPM	Mouse- C57BL/6J	3	1	13	Ambient Temp	21	Whole litter	84	M, F	AFR	EPM	Time spent in open arms (s)	SEM	48.75	6.14	16	64.83	8.72	13	49.29	10.52	7	38.77	5.03	7
Venerosi 2003	Venerosi 2003	Mouse- CD1	3	2	12	Incubator	30	Whole litter	80	M	AFR	EPM	Number of entries into open arms	SEM	1.2	0.25	8	2.91	0.87	9	N/A	N/A	N/A	N/A	N/A	N/A
Xiong 2014	Xiong 2014	Rat- Sprague-Dawley	3	2	12	Ambient Temp	21	Whole litter	56	F	AFR	OFT	% time spent in the center	SEM	N/A	N/A	N/A	N/A	N/A	N/A	14.8	1.78	8	19.54	4.15	9
Veenema 2007	Veenema 2007	Mouse- C57BL/6	3	1	13	Incubator	31.5	Whole litter	84	M, F	AFR	OFT	Time spent in the center (s)	Not Stated	33.07	3.36	18	31.97	2.73	20	43.53	5.21	20	17.98	2.84	18
Romeo 2003	Romeo 2003	Mouse- C57BL/6	3	1	13	Ambient Temp	21	Whole litter	90 Males, 75 Females	M, F	AFR	OFT	Number of center squares crossed	SEM	40.38	6.49	11	27.7	4.06	18	N/A due to estrus cycle					
Tsuda and Ogawa 2012	Tsuda 2012	Mouse- C57BL/6	3	1	13	Incubator	36	Whole litter	91	F	AFR	OFT	Time spent in the center (s)	SEM	N/A	N/A	N/A	N/A	N/A	N/A	161.2	13.86	12	118.07	5.13	23
Bondar 2018	Bondar 2018	Mouse- C57BL/6	3	2	12	Incubator	31	Whole litter	85	M, F	AFR	OFT	Time spent in the center (s)	SEM	9.75	1.51	11	12.19	2.83	6	8.77	1.69	11	10.78	1.96	8
Clinton 2014	Clinton 2014 SD1	Rat- Sprague-Dawley	3	1	13	Incubator	37	Whole litter	75	M	AFR	OFT	% time spent in the center	SEM	5.22	0.84	12	6.9	0.97	12	N/A	N/A	N/A	N/A	N/A	N/A
	Clinton 2014 SD2	Rat- Sprague-Dawley	3	1	13	Incubator	37	Whole litter	75	M	AFR	OFT	% time spent in the center	SEM	2.8	0.66	12	2.14	0.38	12	N/A	N/A	N/A	N/A	N/A	N/A
Shalev 2002	Shalev 2002	Rat- Long Evans	3	3	11	Incubator	32	Whole litter	77	M	NH	OFT	Time spent in the center (s)	SEM	0.26	0.2	15	0.56	0.3	16	N/A	N/A	N/A	N/A	N/A	N/A
Jaimes-Hoy 2016	Jaimes-Hoy 2016*	Rat- Wistar	3	2	19	Incubator	31	Whole litter	40	M, F	AFR	OFT	Time spent in the center (s)	SEM	11	0.4	10	11	1	10	17	2	8	16	2	10
Roman 2006	Roman 2006	Rat- Wistar	6	1	21	Incubator	27	Whole litter	73	M	AFR	OFT	Time spent in the center (s)	SEM	70	6	15	69	10	15	N/A	N/A	N/A	N/A	N/A	N/A
Melo 2018	Melo 2018	Rat- Wistar	3	2	12	Incubator	37	Whole litter	85	M	AFR	OFT	% time spent in the center	SEM	10.07	1.1	7	5.87	0.48	14	N/A	N/A	N/A	N/A	N/A	N/A
Savignac 2011	Savignac 2011 C57	Mouse- C57BL/6	3	1	13	Incubator	N/A	Single	63	M	AFR	OFT	% time spent in the center	SEM	6.5	1.1	15	10.4	1.8	15	N/A	N/A	N/A	N/A	N/A	N/A
	Savignac 2011 BALB/C	Mouse- BALB/c	3	1	13	Incubator	N/A	Single	63	M	AFR	OFT	% time spent in the center	SEM	5.9	1.6	15	4.06	1.1	15	N/A	N/A	N/A	N/A	N/A	N/A
Stevenson 2009	Stevenson 2009	Rat- Lister hooded	6	2	12	Incubator	31	Whole litter	90	F	AFR	OFT	Time spent in the center (s)	SEM	N/A	N/A	N/A	N/A	N/A	N/A	96.8	9.9	11	101.8	4.4	11
Millstein 2007	Millstein 2007 129S1	Mouse- 129S1/SvImJ (129	3	0	13	Incubator	32	Single	56	M, F	AFR	OFT	% time spent in the center	SEM	3.96	2.23	12	13.35	10.85	9	1.47	0.73	13	9.47	4.4	15
	Millstein 2007 BALB/c	Mouse- BALB/cByJ	3	0	13	Incubator	32	Single	56	M, F	AFR	OFT	% time spent in the center	SEM	9.88	1.95	13	18.36	3.06	15	16.44	3.18	15	15.65	2.81	13
	Millstein 2007 C57	Mouse- C57BL/6J	3	0	13	Incubator	32	Single	56	M, F	AFR	OFT	% time spent in the center	SEM	18.08	1.81	12	15.86	1.67	13	13.45	2.44	16	18.09	1.96	11
	Millstein 2007 DBA	Mouse- DBA/2J	3	0	13	Incubator	32	Single	56	M, F	AFR	OFT	% time spent in the center	SEM	15.23	3.2	15	5.01	1.81	10	14.3	11	9	20.42	7.33	18
	Millstein 2007 FVB	Mouse- FVB/NJ	3	0	13	Incubator	32	Single	56	M, F	AFR	OFT	% time spent in the center	SEM	22.95	1.95	11	15.99	1.25	18	17.54	1.34	18	19.99	1.71	13
Jin 2018	Jin 2018	Rat- Wistar	3	1	20	Not stated	N/A	Not stated	45	M, F	AFR	OFT	Number of entries into the center	SD	4.6	1.27	8	2.45	0.87	8	4.4	1.42	14	1.99	1.42	14
Nam 2014	Nam 2014	Rat –WKY	3	1	13	Incubator	37	Whole litter	75	M	AFR	OFT	Time spent in the center (s)	SEM	7.5	3.62	15	7.35	2.03	15	N/A	N/A	N/A	N/A	N/A	N/A
Shu 2015	Shu 2015	Rat- Sprague-Dawley	3	2	12	Ambient Temp	21	Whole litter	90	M	AFR	OFT	Distance traveled in the center (cm)	SD	487.05	326.47	12	196.9	85.49	12	N/A	N/A	N/A	N/A	N/A	N/A
Ershov 2018	Ershov 2018	Mouse- C57BL/6	3	2	14	Incubator	31	Whole litter	95	M	AFR	OFT	Time spent in the center (s)	SEM	9.77	1.62	11	12.22	2.79	6	N/A	N/A	N/A	N/A	N/A	N/A
Aya-Ramos 2017	Aya-Ramos 2017	Rat- Wistar	6	1	21	Not stated	N/A	Not stated	51	M, F	AFR	OFT	% time spent in the center	SEM	4.86	1.8	10	8.45	1.91	10	7.59	1.52	8	3.46	0.67	7
Kundakovic et al. 2013	Kundakovic et al. (2013)	Mouse- Balb/C	2	1	13	Not stated	N/A	Not stated	35	M, F	AFR	OFT	Time spent in the center (s)	SEM	82.78	15.19	10	65.81	8.34	10	48.83	11.02	10	63.42	11.02	10
Tsuda 2014	Tsuda 2014	Mouse- C57BL/6J	4	1	13	Incubator	36	Whole litter	91	M, F	AFR	OFT	Time spent in the center (s)	SEM	165.93	19.91	9	126.77	18.58	6	233.99	28.62	7	157.7	12.84	7
Pierce 2014	Pierce 2014 14	Mouse- C57BL/6	3	1	14	Incubator	34	Whole litter	42	F	AFR	OFT	% time spent in perimeter	SEM	N/A	N/A	N/A	N/A	N/A	N/A	84.6	3.69	6	82.85	7.66	5
	Pierce 2014 21	Mouse- C57BL/6	3	1	21	Incubator	34	Whole litter	42	F	AFR	OFT	% time spent in perimeter	SEM	N/A	N/A	N/A	N/A	N/A	N/A	59.13	3.86	7	36.76	5.53	8
Own 2013	Own 2013	Mouse- C57BL/6	3	2	12	Incubator	31	Whole litter	70	M	AFR	OFT	Time spent in the center (s)	SEM	23.9	1.3	30	24.3	1	29	N/A	N/A	N/A	N/A	N/A	N/A
Diehl 2007	Diehl 2007	Rat- Wistar	3	1	9	Incubator	34	Whole litter	88	M, F	NH	OFT	Time spent in the center (s)	SEM	9.05	3.1	12	6.14	0.96	12	5.18	1.69	12	7.99	1.69	12
Weiss 2011	Weiss 2011 OFT	Mouse- C57BL/6J	3	1	13	Ambient Temp	21	Whole litter	84	M, F	AFR	OFT	Time spent in the center (s)	SEM	40.96	9.24	8	70.28	12.65	6	47.66	13.23	6	52.35	6.38	7
Markostamou 2016	Markostamou 2016	Rat- Wistar	3	1	5	Incubator	31	Whole litter	90	M, F	AFR	OFT	Time spent in center (s)	SD	39.8182	17.67381	11	59.5	2.12132	2	41	17.39732	7	30.1667	14.8784	6
